# Impact of mandatory vaccination against serogroup C meningococci in targeted and non-targeted populations in France

**DOI:** 10.1038/s41541-022-00488-8

**Published:** 2022-06-29

**Authors:** Samy Taha, Muhamed-Kheir Taha, Ala-Eddine Deghmane

**Affiliations:** 1 Institut Pasteur, Invasive bacterial infections Unit and National Reference centre for meningococci and Haemophilus influenzae, Paris, France; 2 Université de Paris Sud, Faculty of Medicine, Le Kremlin-Bicêtre, France

**Keywords:** Meningitis, Meningitis

## Abstract

Since January 2018, mandatory vaccination against meningococci serogroup C has been implemented in France for children <2 years with a recommended catch-up vaccination until the age of 24 years. We aimed to analyse the impact of mandatory vaccination on populations not targeted by it (2–24 years old). We used the database of the national reference centre for meningococci to collect the number of invasive meningococcal disease (IMD) cases before (2016–2017) and after (2018–2019) the mandatory vaccination. The cultured isolates were sequenced and submitted for genomic comparison. The total number of cases was 1706, including 376 cases of IMD serogroup C. Mandatory vaccination correlated with a significant decrease among the <2 years old and a decreasing trend among the 2–14 years old group but not among 15–25 years of age. This observation may be explained by the vaccine coverage that is still low among adolescents and young adults. Moreover, the genomic analysis revealed the co-circulation of two major genotypes belonging to the clonal complex ST-11 whose distribution differed across the age groups in accord with cyclic variations of genotypes. It is important to increase specific knowledge on meningococcal epidemiology and vaccination to involve them in establishing the vaccination strategy.

## Introduction

Invasive meningococcal disease (IMD) is a severe rapidly evolving invasive infection provoked by *Neisseria meningitidis* (Nm) that is usually carried asymptomatically in the nasopharynx of ~10% of the general population^1^. However, invasive isolates of *N. meningitidis* are usually encapsulated and invade the bloodstream to provoke severe systemic infections that are dominated by septicaemia and meningitis as well as other non-meningeal forms. The capsular polysaccharides determine the serogroup of the isolates among the 12 known serogroups of which 6 are responsible for virtually all IMD cases worldwide (A, B, C, W, X and Y)^2^. Moreover, the isolates can be typed genetically on the basis of sequencing of several chromosomal loci (multi-locus sequence typing, MLST) into sequence type (ST) that corresponds to the combination of the allelic profile of the sequenced loci^3^. Close STs can further be grouped into Clonal Complexes (CC). Whole-genome sequencing (WGS) is also used for typing and allows extraction of MLST data and provides more resolution among isolates^4^.

If untreated, IMD is almost always fatal but the antibiotic era marked a huge improvement in the management of IMD with a drastic drop in the fatality rate. However, even treated it still shows a high fatality rate of 10% and up to 20% of survivors suffer permanent disabling sequelae that impair their quality of life^5–7^. Recognition of IMD at an early stage is still difficult as early symptoms and signs are usually non-specific and atypical forms may delay the management. Moreover, reduced or non-susceptibility to beta-lactams, the key antibiotic used in the treatment of IMD, is increasingly reported at the global level^2^ and IMD due to non-susceptible isolates has been reported^8^. Prevention of IMD is therefore a key element in reducing the morbidity and mortality of IMD. Vaccination, an efficient prevention strategy against IMD, and vaccines against *N. meningitidis* are available against 5 of the six most IMD-associated serogroups. Conjugate monovalent and quadrivalent capsular polysaccharide-based vaccines are available against serogroups A, C, W and Y. Protein-based vaccines are licensed against serogroup B. Different strategies were tailored to implement these vaccines across the world. In France, vaccination against meningococci C, using meningococcal C conjugate (MCC) vaccines, was recommended in 2010 for toddlers in the second year of life and a catch-up up to the age of 24 years. However, vaccination coverage remained low in particular among 16–24 years of age and there was also an increase in the number of new cases among those under 1 and over 25^9^. This observation promoted the introduction in 2017 of an additional dose at 5 months of age and in 2018 the strategy was replaced by the mandatory vaccination with two doses at 5 and 12 months of age with MCC and the catch-up campaign up to 24 years of age remained recommended. The other vaccines against serogroups (ACWY) and (B) are only recommended for at-risk subjects and for the control of outbreaks.

One year after the implementation of mandatory vaccination that included 11 vaccines for children under 2 years old, vaccination has significantly increased in these children and an increasing trend was also observed for other vaccines not concerned by mandatory vaccination^10^. However, no observations are yet available for the impact on other groups of age and in particular for MCC for subjects between 2 and 24 years of age. We aimed to this effect by using the proxy of IMD cases among 2–24 years old before and after the implementation of mandatory vaccination. We searched for a significant difference in the number of cases and a correlation, if any, with the vaccine coverage in this group.

## Results

### General characteristics of IMD cases during the study period

We first analysed all of the IMD cases in the database (*n* = 1737), over the four years (2016–2019) covered by our study. M:F ratios and age distribution of cases were not significantly different from 1 year to another over the duration of the study (Table 1).

**Table 1:** General characteristics of IMD cases during the period 2016–2019.

Year		2016	2017	2018	2019
Number of cases		450	474	397	416
Sex ratio M:F		1.03	0.88	0.88	0.79
Age distribution (%)	<2 years	21	18	19	23
	2–24 years	37	30	34	33
	≥25 years	42	52	47	44
Serogroup distribution N° (%)	B	224 (50)	199 (42)	195 (49)	214 (51)
	C	113 (25)	122 (26)	88 (22)	53 (13)
	W	47 (10)	70 (15)	57 (14)	88 (21)
	Y	61 (14)	75 (16)	55 (14)	52 (13)
	Others	5 (1)	8 (1)	2 (1)	9 (2)

Serogroup distribution showed variations according to the four major serogroups (B, C, W and Y). Serogroup B was the most prevalent and remains stable with 224 cases in 2016, 199 cases in 2017, 195 cases in 2018 and 214 cases in 2019 (Table 1). Serogroup Y also remains stable with 61 cases in 2016, 75 cases in 2017, 55 cases in 2018 and 52 cases in 2019. Serogroup C decreased by 113, 122, 88 and 53 cases respectively from 2016 to 2019. Serogroup W increased and the number of cases almost doubled in 4 years, increasing from 47 cases in 2016 to 88 cases in 2019, with 70 cases in 2017 and 57 cases in 2018 (Table 1).

### IMD serogroup C cases since the implementation of mandatory vaccination

**Fig. 1:**
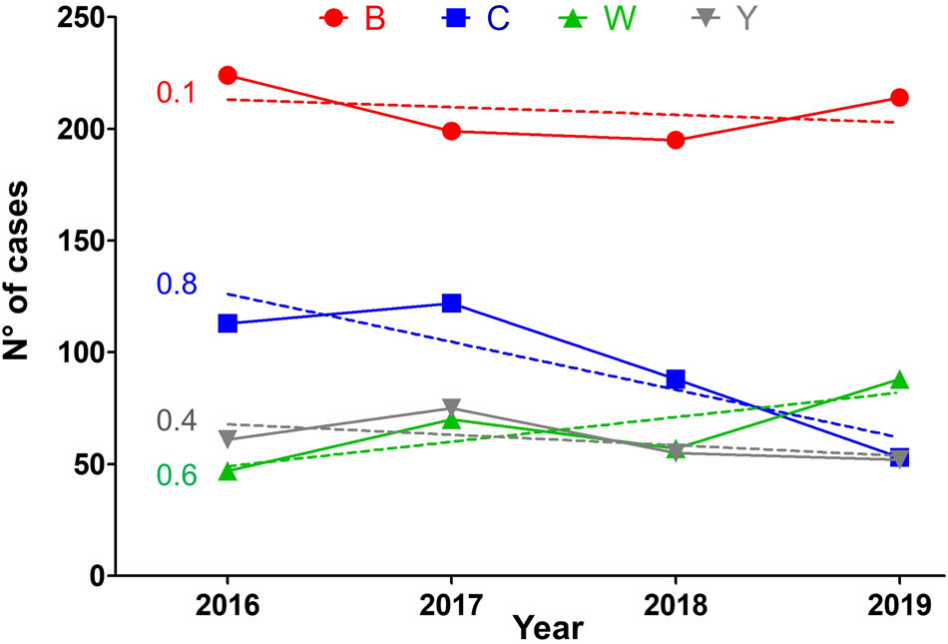
Evolution of number of cases of IMD per serogroup during the period 2016–2019. The four serogroups (B, C, W and Y) are represented by a different colour with the number of cases indicated per year. The dashed lines represent the linear regression for the corresponding serogroup with the *r*^2^ value indicated with the same colour as the corresponding serogroup.

**Fig. 2:**
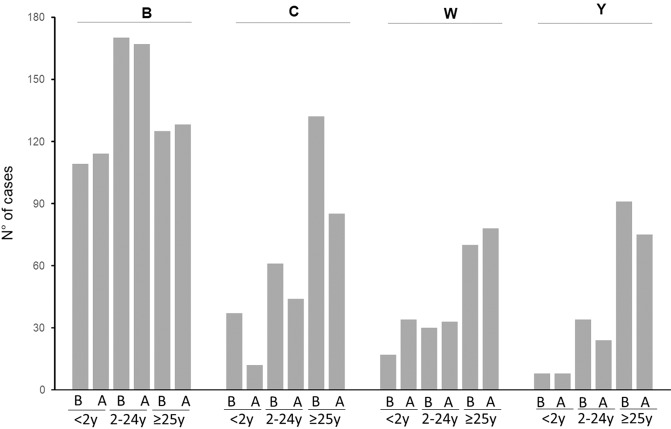
Distribution of cases of IMD per serogroup and per age group during the period 2016–2019. Three age groups are shown (<2 years, 2–24 years and ≥25 years). Data per period before compulsory vaccination (**B**: 2016–2017) and after (**A**: 2018–2019) are shown.

**Fig. 3:**
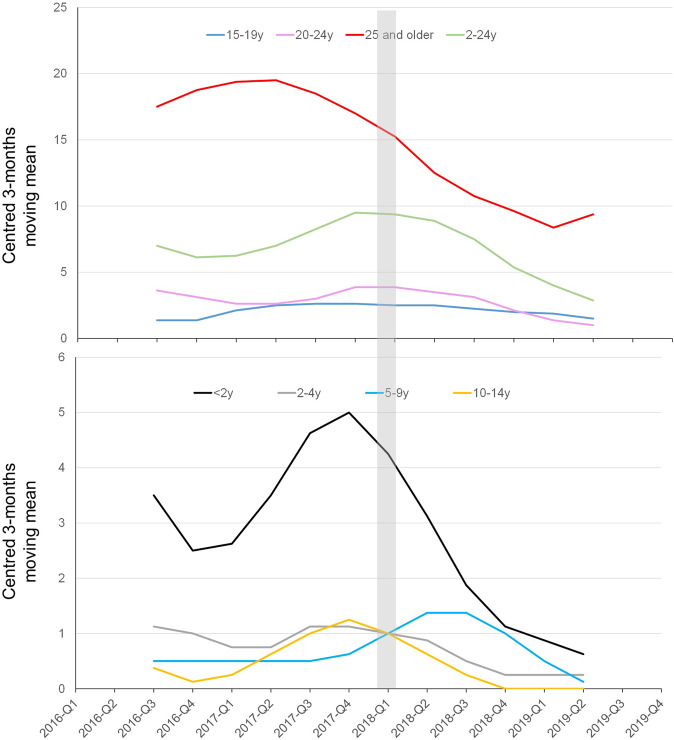
Evolution of a number of cases of IMD due to serogroup C per age group during the period 2016–2019. Time-series analysis was used with the data expressed per quarter of each studied year. The data were expressed by the centred 3-months moving mean per age group. Age groups are indicated in different colours with the corresponding number of cases per year. The date of the implementation of mandatory vaccination is indicated by a grey box.

The total of cases of serogroup C for the studied period (2016–2019) was 376 cases among which 332 (88%) cases were confirmed by culture and 44 (12%) cases were confirmed by PCR only. The percentage of cases that were only confirmed by PCR was stable over the whole period and was 10%, 13%, 11%, and 13% for 2016, 2017, 2018, and 2019, respectively. A global drop of 40% IMD of serogroup C (IMDC) was observed since 2018 (total cases of 235 for both 2016 and 2017 versus 141 for 2018–2019). Variations were also observed for other serogroups over this period but were less important (3% drop for IMDB, 24% increase for IMDW and 21% drop for IMDY). However, these trends did not reach significant levels (Fig. 1). Moreover, IMD cases due to serogroups B, W and Y combined together (IMDBWY), remained stable for the same periods (676 cases for the period 2016–2017 versus 661 for the period 2018–2019 with 1% reduction). We therefore analysed these variations for the four serogroups according to age groups that were targeted by mandatory vaccination (<2 years), recommended vaccination (2–24 years) and those with no recommendation (≥25 years). IMDC cases were compared to IMD due to serogroups B, W and Y (Table 1). IMDC cases showed decreasing trend for the three age groups. However, only the trend in <2 years showed a slope with a significant deviation from zero (*P* = 0.00057). This significant decrease persisted after distributing the number of cases IMDC and IMD of the three other serogroups (B, W and Y) over the whole 4 years period into “before” and “after” mandatory vaccination groups and for the three age groups (Fig. 2). We therefore further analysed the 2–24 years population in more details by comparing the groups <2 years, 2–4 years, 5–9 years, 10–14 years, 15–19 years and 20–24 years for IMDC. We also added the group of 25 years and older who were not targeted by the vaccination. Time-series analysis showed decline trends in all age groups <2 years, 2–4 years, 5–9 years and 10–14 years although the centred 3-months means were low for the last three age groups. These decreases were observed since the first quarter of 2018 or later (Fig. 3). This decrease was also observed when the combined group of 2–24 years was considered (Fig. 3). This trend was not observed for the 15–19 years and 20–24 years old groups. However, a dressing trend was observed in the group 25 years old and older but that started in the second quarter of 2017 prior to the implementation of the mandatory vaccination (Fig. 3).

### Correlation between IMDC cases and the evolution of vaccine coverage rates

**Fig. 4:**
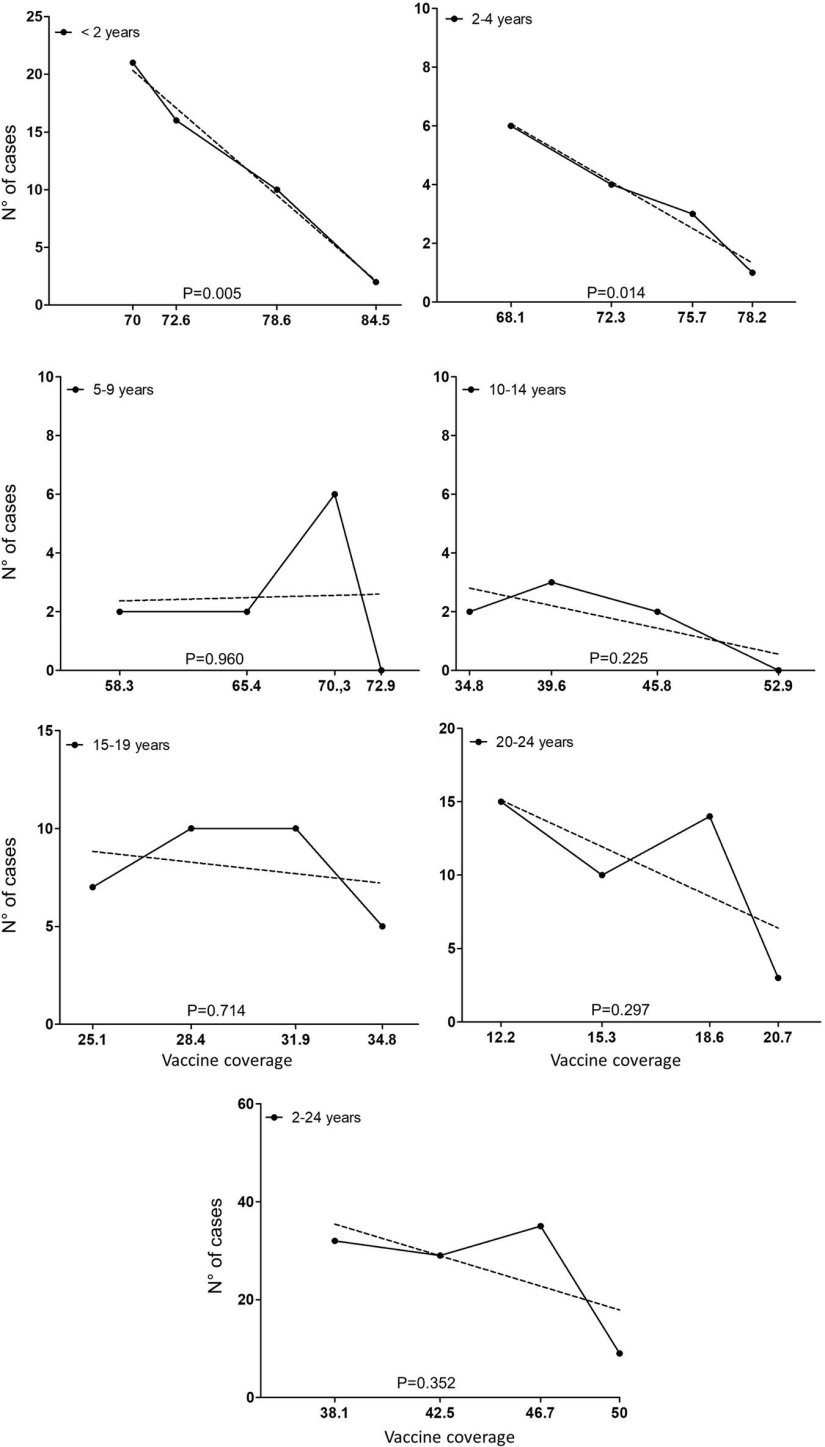
Evolution of number of cases of IMD due to serogroup C per age group according to vaccine coverage. Age groups are indicated. The number of cases is indicated on the vertical axis. The horizontal axis represents the coverage rate (in percentage) per year from 2016 (fat left) to 2019 (far right) and linear regression (dashed lines). *P* values for a slope different from zero are shown.

We next looked for a correlation between the number of IMDC cases and vaccination coverage by performing linear regressions between the number of cases and the vaccine coverage rates across the period 2016–2019. The analysis was performed on the same age groups: (<2 years, 2–4 years, 5–9 years, 10–14 years, 15–19 years, and 20–24 years). As expected, in children under 2 years of age, there is a statistically significant correlation between the number of IMDC cases and vaccination coverage (*p* = 0.005). A statistically significant correlation was also found between the number of IMDC cases and vaccination coverage in 2–4 year olds group (*p* = 0.014). However, no significant correlation was found for the other age groups. No significant correlation was observed when the whole age band (2–24 years) was considered as a single group (Fig. 4).

### Genomic characteristics of culture-confirmed cases

**Fig. 5:**
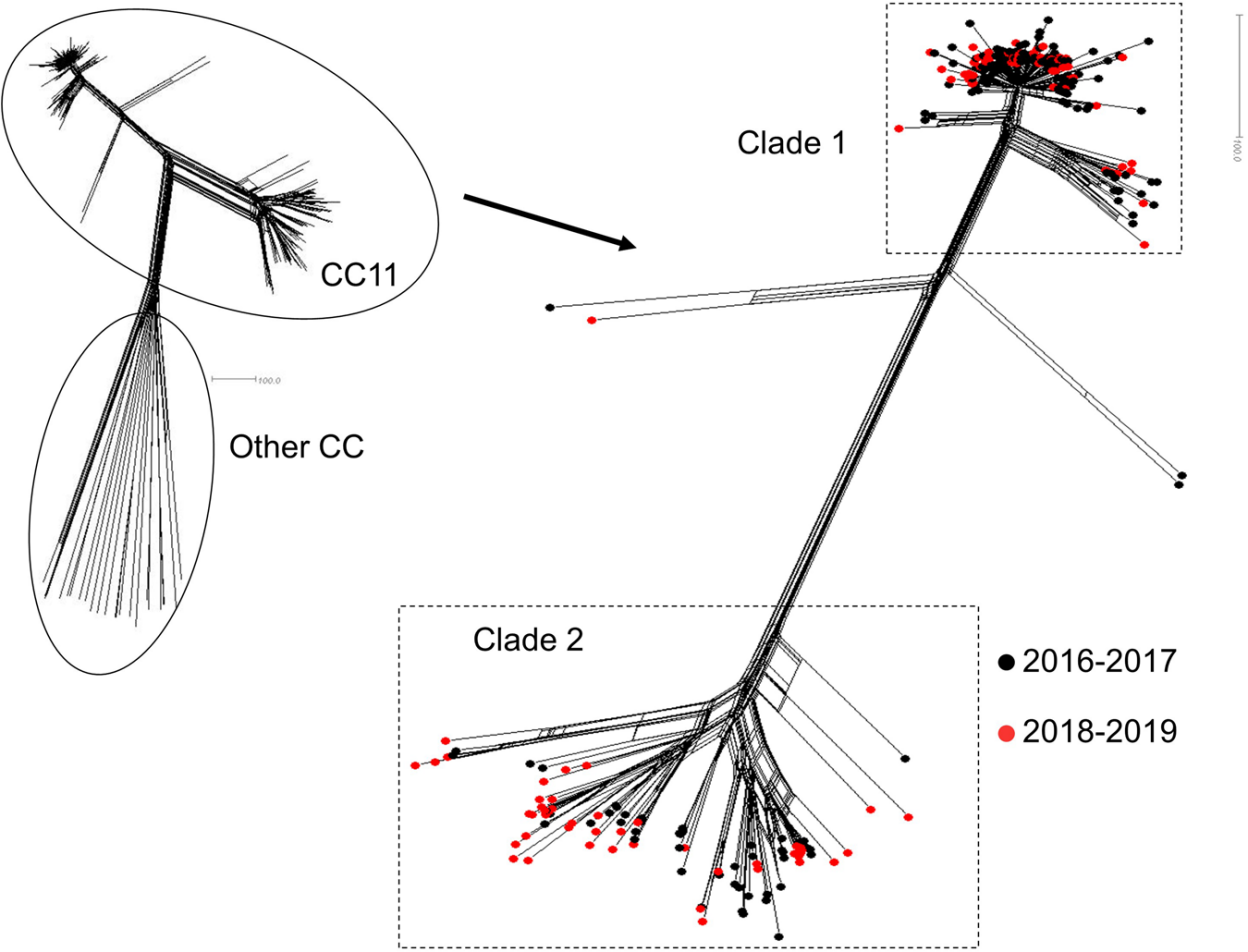
Neighbour-net phylogenetic network of all IMD culture-confirmed cases due to NmC cases in France for the period 2016–2019. The major genotype (CC11) is indicated. To the right, the two major Clades (1 and 2) of the CC11 are surrounded by dashed lines. Isolates from the two periods 2016–2017 (before the mandatory vaccination) and 2018–2019 (after the mandatory vaccination) are indicated in black and red circles respectively.

The decrease of IMDC cases in the group aged 25 years and older in the absence of vaccine recommendations for this group prompted the analysis towards the exploration of the genomic structure of NmC isolates. Among the 332-culture-confirmed cases (88% of all IMDC), we successfully recovered by culture, 313 isolates (94% of culture-confirmed cases and 83% of all the 376 IMDC cases) and obtained WGS data by using next-generation sequencing (See Materials and Methods and supplementary Table 1). MLST extracted from WGS data showed that the vast majority of these 313 isolates belonged to hyperinvasive clonal complex CC11 (*n* = 290; 93%). A gene-by-gene cgMLST was performed on 1605 genes and allowed to draw the network tree depicted in Fig. 5. The analysis of this tree showed that 286 of the 290 CC11 isolates were distributed into two major clades named clade 1 and clade 2 (Fig. 5) and were composed of 190 and 96 isolates respectively. Interestingly, the isolates of clade 1 shared the genotypic formula of C:P1.5,2:F3-3:CC11 while the isolates of clade 2 shared the formula C:P1.5-1,10-8, F3-6:CC11.

**Fig. 6:**
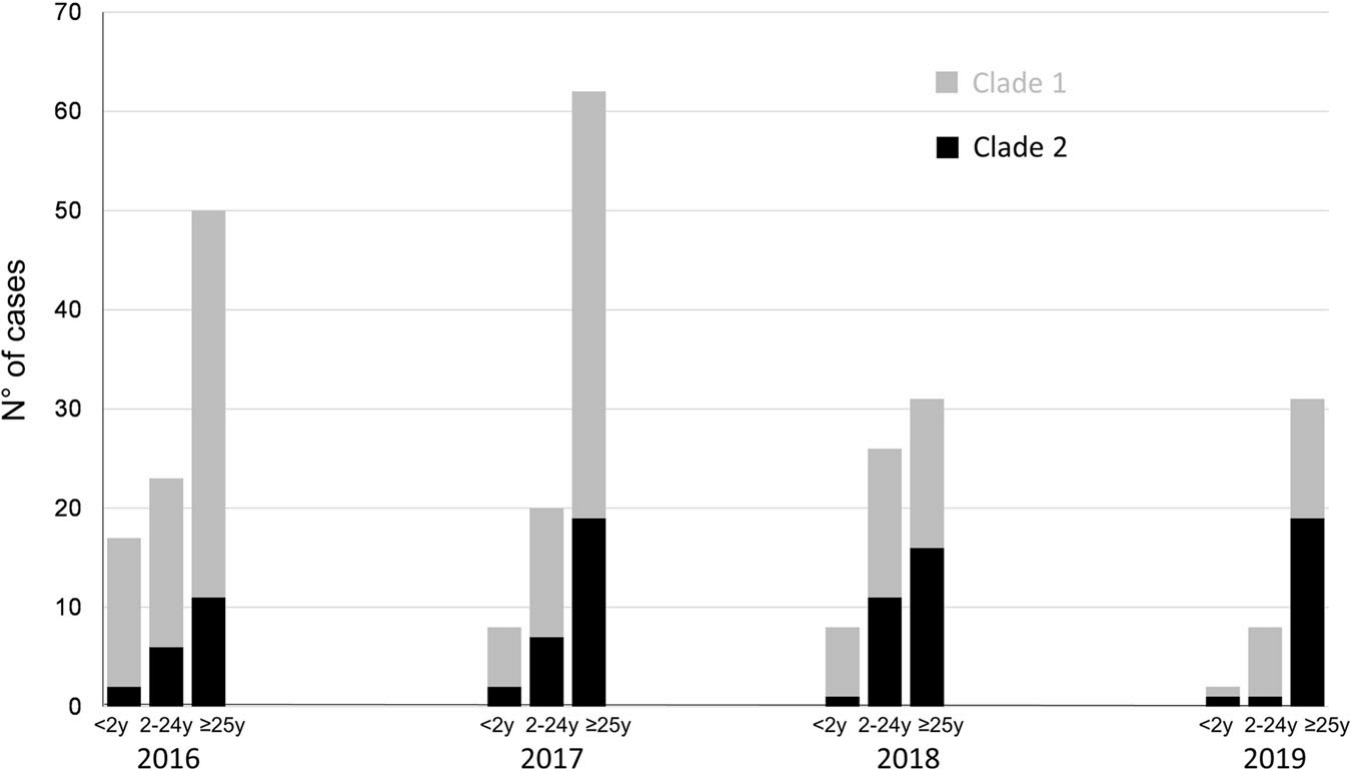
Distribution of cases of IMD duo to serogroup C per age group during the period 2016 (far left) to 2019 (far right). Three age groups are shown for each year (<2 years, 2–24 years and ≥25 years). Data show the number of isolates of Clade 1 and Clade 2 per age group and per year per.

Isolates of clade 1 were more prevalent than clade 2 all over the period of the study and in each individual year. However, clade 1 drastically decreased (from 71 in 2016 case to 20 cases in 2019, *P* = 0.01) while isolates of clade 2 remained almost stable over the whole 4 years period (19 isolates in 2016 and 21 in 2019, *P* = 0.83) (Fig. 6). Isolates of clade 1 have decreased among <2 years group as well as in the group of 2–24 years old but also in the group of ≥25 years old, that was not targeted by the vaccination. Isolates of clade 2 remained stable among the 25 years and older group although it showed a slight decrease in the groups of <2 years and 2–24 years. These data suggest that periodic changes in the genotypes of meningococcal isolates may also contribute to the observed evolution in the number of cases among different age groups.

## Discussion

The compulsory vaccination in France implemented in 2018 had a significant effect on the number of cases of all infections covered by the 11 vaccines made compulsory in the targeted populations (<2 years old)^10^. This compulsory vaccination included vaccination against meningococci C at the age of 5 months and 12 months for all infants born since 1 January 2018. Our data confirm the effect of compulsory vaccination in <2 years of age with a significant drop in the number of cases of IMDC since 2018, absent among other serogroups, with a significant increase for serogroup W. Moreover, a significant correlation between the number of IMDC cases and MCC vaccination coverage rate in <2 years. Indeed, an increase in the vaccine coverage rates in this group to 84.5% in 2019 was observed. Only two cases of IMDC in this age group were reported to the NRCMHi in 2019. Vaccination against NmC also maintained a catch-up recommendation among 2–24-year olds in parallel to the compulsory vaccination in the <2 years. However, our study did not find a decreasing trend beyond the age of 14 years. Indeed, the vaccine coverage remains suboptimal, particularly among subjects 15–24 years old, the primary source of *N. meningitidis* transmission to other age groups^9^. Moreover, the vaccine coverage rates in this group do not consider the time since vaccination. This information is important as the persistence of protective bactericidal titres is lower when the MCC is administered in early childhood^11^. This observation warrants analysing the data of the 2–24 years old according to several subgroups (<2 years, 2–4 years, 5–9 years, 10–14 years, 15–19 years and 20–24 years). The decreasing trends that were observed among the groups of 2–4 years, 5–9 years and 10–14 years would be in favour of a direct effect of the implementation of vaccination since 2010, the introduction in 2017 of the dose at 5-months of age and vaccination obligation in <2 years old since 2018. The shift over time of the cohorts towards the groups of 2–4 years, 5–9 years and 10–14 years groups revealed this effect. Furthermore, given the highest rates of meningococcal carriage among adolescents, the primary reservoir/transmitter to other age groups, their vaccination has been proposed as an efficient strategy if high coverage can be achieved^12,13^. In our study, the low decline in the number of IMDC cases among the other age groups correlated with the modest coverage rate observed in adolescents.

However, our data revealed a decreasing trend in the number of cases in the group of 25 years and older although this group was not targeted by the vaccination strategy against IMDC. This decreasing trend may be linked to other factors that can influence the evolution of IMD cases such as the seasonal flu due to the spatiotemporal association between flu and IMD^14^. Additionally, periodic variation can be observed independently of any health intervention and can be linked to the evolution of different circulating genotypes due to new combinations of alleles arising from recombination, thus pertaining to new profiles^15^. Our genomic analysis suggests that two major genotypes co-circulate during the period 2016–2019. The most prevalent genotype (Clade 1) seems to have undergone a reduction in the number of cases in all age groups including the 25 years and older group that is not targeted by the vaccination. This genotype was involved in the hyperendemic cases of IMDC in 2011 in the region of Britany, France^16^. The second genotype (Clade 2) was reduced in groups targeted by the vaccination (mandatory among <2 years and recommended among 2–24 years). This genotype was responsible for the outbreak among men who have sex with men in France and Germany in 2013^17^.

A high vaccination coverage rate is a major public health indicator. However, reaching and maintaining such a target for public health institutions is challenging This task is further complicated by the high level of vaccine refusal and hesitancy in France^18^. Compulsory vaccination can be a tool to overcome the high vaccination hesitancy in France. Indeed, the direct consequences were significant among <2 years. However, our data suggest that the indirect benefits were limited, if any, in other age groups. Other European countries achieved a high level of vaccine coverage (>90%) without compulsory vaccination^19^.

The main limitation of our study is the lack of power, due to a low number of cases in several age groups, and the short period since the introduction of mandatory vaccination. However, the incidence of IMD decreased drastically since the COVID-19 pandemic most likely due to social and physical distancing measures^20,21^. This situation hinders studies covering longer periods after the implementation of compulsory vaccination against IMD. Surveillance of IMD cases, their age distribution and genotypes of the isolates as well as correlation with vaccine coverage is therefore crucial.

The general practitioner is the first point of contact for patients, in particular for aspects related to vaccination. Key messages for the vaccination against NmC can be delivered such as the information that the dose introduced at 5 months, although necessary, remains insufficient as long as the vaccination coverage remains low. Even with the two doses at 5 and 12 months, the duration of protective immune response is limited due to the waning of bactericidal titres^11^. Intensive catch-up is crucial to establish both individual and herd immunity among 2–24 years old group. Due to the failure in the implementation of a successful catch-up campaign in France^11^, a booster dose of MCC vaccine in adolescence can also be considered to keep the young adults protected when subjects who were vaccinated at an early age reach adulthood. The quadrivalent ACWY vaccine can also be considered due to the increase in IMDW.

## Methods

### Data sources

**Fig. 7:**
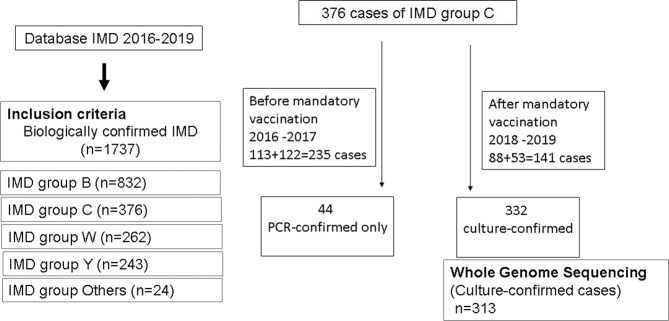
Study flowchart. IMD: invasive meningococcal disease.

Surveillance of IMD in France is based on two components: (i) mandatory reporting of cases to regional health agencies (ARS) and Public Health France (SpF) according to a standardised national definition of cases^9^ and (ii) the typing of isolates at the National Reference Centre for meningococci and *Haemophilus influenzae* (NRCMHi) isolates as institutional missions of the NRCMHi. Both sources are matched anonymously using a common anonymous code generated for each case and the exhaustiveness of the mandatory notification of IMD cases system has been > 90% since 2005^9^. We extracted the data from the NRCMHi database for all IMD cases for the period of two years 2016–2017 and for the period of two years 2018–2019 (before and since the mandatory vaccination respectively) (Fig. 7).

We collected information on age, sex, date of onset of IMD, and the group of the isolates. All ages were extracted for global analysis but further evaluation of the evolution of IMD related to serogroup C (IMDC) focused on the following groups of age: <2 years, 2–4 years, 5–9 years, 10–14 years, 15–19 years and 20–24 years, for which data on vaccination coverage by age group and by year were available from SpF^22^.

### Genetic typing and whole-genome sequencing

PCR-confirmed cases were typed by MLST analysis and all cultured isolates were typed by WGSusing Illumina® technology of next-generation sequencing^9^. Briefly, genomic DNA was extracted from cultured isolates with the MagNA Pure 96 system (Roche Molecular System, Pleasanton, USA). Library preparation was performed with the Nextera^®^ XT DNA library Preparation Kit (Illumina, San Diego, USA) and WGS was processed with Illumina^®^ technology (NextSeq 500, Illumina) with paired-end strands of 150 bp and a sequencing depth of 50×. All de novo assemblies were performed with SPAdes (CAB, St. Petersburg State University, Russia). Typing data were expressed as a genetic formula (g:P1.PorA-VR1,PorA-VR2:FetA:CC) that defines the group (g), the two variable regions (VR1 and VR2) of the outer membrane protein PorA, and of the VR of the protein FetA, as well as the CC. These characteristics were extracted from WGS. Relationships between the isolates were analysed using a “gene-by-gene” approach of the core genome MLST (cgMLST v1.0) through the BIGSdb Genome Comparator tool available in PubMLST platform^23^. SplitsTree4 (version 4·13·1) was used to visualise the resulting distance matrices as Neighbour-net networks^24^. The identification numbers (id) of all cultured isolates are given in Supplementary Table 1 to allow retrieving of WGS sequence in FASTA formats from the PubMLST website.

### Statistical analysis

Statistical analyzes were performed using GraphPad PRISM 5.0.1 software. Descriptive analysis of the number of cases, age, sex and serogroup distribution by year was conducted. Chi-square tests were used to compare the number of cases before and after the implementation of mandatory vaccination. The significance level is a *P* value <0.05 for single comparisons. Time series were used to express the evolution of a number of IMDC cases per quarter year and per age group (<2 years, 2–4 years, 5–9 years, 10–14 years, 15–19 years 20–24 years that are targeted by the vaccination. We also added a combined group 2–24 years old in addition to the group of 25 years and older who are not targeted by the vaccination. Linear regressions and correlation tests by age group were used to measure the trend of the evolution of the number of cases and the correlation between the number of IMDC cases and vaccination coverage year by year.

### Reporting summary

Further information on research design is available in the Nature Research Reporting Summary linked to this article.

## Supplementary information

Supp Table 1

Reporting Summary

## Data Availability

Genomic data are available on https://pubmlst.org/neisseria/. Anonymized individual data that support the findings of this study are available on request from the corresponding author [M.K.T.].
